# The new NCPSS BL19U2 beamline at the SSRF for small-angle X-ray scattering from biological macromolecules in solution^1^

**DOI:** 10.1107/S160057671601195X

**Published:** 2016-08-10

**Authors:** Na Li, Xiuhong Li, Yuzhu Wang, Guangfeng Liu, Ping Zhou, Hongjin Wu, Chunxia Hong, Fenggang Bian, Rongguang Zhang

**Affiliations:** a National Center for Protein Science Shanghai, Shanghai Institutes of Biological Sciences, No. 320, Yueyang Road, Shanghai, 200031, People’s Republic of China; b Shanghai Synchrotron Radiation Facility, Shanghai Institutes of Applied Physics, No. 239, Zhangheng Road, Shanghai, 201204, People’s Republic of China

**Keywords:** biological small-angle X-ray scattering, automation and high brilliance, structural biology

## Abstract

The beamline BL19U2 is located in the Shanghai Synchrotron Radiation Facility (SSRF) and is its first beamline dedicated to biological material small-angle X-ray scattering (BioSAXS). The electrons come from an undulator which can provide high brilliance for the BL19U2 end stations. A double flat silicon crystal (111) monochromator is used in BL19U2, with a tunable monochromatic photon energy ranging from 7 to 15 keV. To meet the rapidly growing demands of crystallographers, biochemists and structural biologists, the BioSAXS beamline allows manual and automatic sample loading/unloading. A Pilatus 1M detector (Dectris) is employed for data collection, characterized by a high dynamic range and a short readout time. The highly automated data processing pipeline *SASFLOW* was integrated into BL19U2, with help from the BioSAXS group of the European Molecular Biology Laboratory (EMBL, Hamburg), which provides a user-friendly interface for data processing. The BL19U2 beamline was officially opened to users in March 2015. To date, feedback from users has been positive and the number of experimental proposals at BL19U2 is increasing. A description of the new BioSAXS beamline and the setup characteristics is given, together with examples of data obtained.

## Abstract 2

A new biological small-angle X-ray scattering beamline (BioSAXS, BL19U2) at the Shanghai Synchrotron Radiation Facility (SSRF) is dedicated exclusively to small-angle scattering experiments from biological macromolecules in solution. As part of the important facilities in the National Center for Protein Sciences Shanghai (NCPSS), this BioSAXS beamline is the first in China to serve the rapidly increasing biology communities.

## 1. Introduction   

Small-angle X-ray scattering (SAXS) has been employed for a long time in polymer and materials science (Guinier, 1969 ▸), and its popularity in biology has experienced a steady growth attributed to the availability of synchrotron sources and the novel data analysis methods developed specifically for biological macromolecules in solution (Svergun & Koch, 2003 ▸; Nagar & Kuriyan, 2005 ▸; Petoukhov *et al.*, 2012 ▸). As such, biological small-angle X-ray scattering (BioSAXS) now plays an important complementary role in molecular and structural biology and gives rise to a continually growing interest within the biological community (Jacques & Trewhella, 2010 ▸). The beamline BL19U2 is the newly constructed beamstation for SAXS experiments on biological materials in solution at the Shanghai Synchrotron Radiation Facility (SSRF). As an important part of the Chinese National Centre for Protein Science Shanghai (NCPSS) construction programme (http://www.ncpss.org/sheshiOver.action?summaryId=4), it satisfies the growing demand for SAXS from biological communities in China.

The decision to build the BL19U2 beamline of the NCPSS, located on an undulator of the SSRF storage ring, was taken in 2011. It took three years to carry out the major renovation, and pilot runs of the beamline began in September 2014. It was officially opened to users in March 2015. In considering its specificity in biological macromolecule studies, the design of this beamline enhanced its stability; an automated sample changer was installed for reliable SAXS experiments in solution. Access to the BioSAXS beamline is either through the NCPSS beamtime application system (http://www.ncpss.org/userGuideApp.action) or through a rolling application system which accepts proposals at any time. To date, feedback from users has been positive and the number of experimental proposals at BL19U2 is increasing, showing that the BioSAXS facility of BL19U2 at the SSRF is already providing reasonable SAXS results.

## 2. Beamline overview   

The beamline BL19U2 of the NCPSS is from an undulator source, which shares one straight section with another crystallography beamline (BL19U1), allowing two different user groups to collect data simultaneously. Two undulators, providing high brilliance and energy tunability between 7 and 15 keV for both end stations (BL19U1 and BL19U2), are canted by 6 mrad to give enough horizontal beam separation between the two beamlines. The RMS source size is 380 µm [horizontal (H)] × 25 µm [vertical (V)] and the divergence is 80 µrad (H) × 30 µrad (V). Both BL19U1 and BL19U2 beamlines make use of the small gap undulator U20 as the source, providing a compromise between the brightness and the wavelength tunability. As a result, changing the energy at station BL19U1 does not lead to a change in flux at BL19U2. This offers the possibility for automated operation and remote-access data collection.

### 2.1. Optical elements   

**Figure 1:**
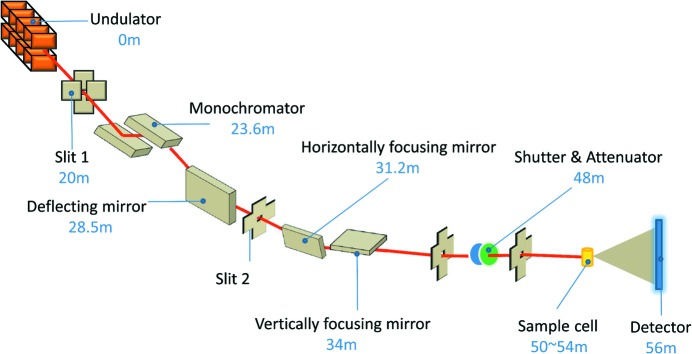
Optical layout of the BL19U2 BioSAXS beamline with the source-to-main-element distances.

The *Bremsstrahlung* collimator was installed at the exit of the front end to narrow the dimensions of *Bremsstrahlung* radiation. White-beam slits located 20 m from the beam source are used to define the white-beam profile (Fig. 1 ▸). The beamline BL19U2 has a maximum acceptance cone of 0.08 mrad (H) × 0.05 mrad (V). The monochromator is 3.6 m downstream of the white-beam slits. A vacuum differential pumping system was used to evacuate the different vacuum sections in front of the monochromator. Retractable polycrystalline diamond fluorescence screens, for use in the monochromatic beam, were installed, allowing easy beam alignment and diagnostics. An additional *Bremsstrahlung* collimator was installed after the monochromator to further narrow the dimensions of *Bremsstrahlung* radiation. Beamline details are summarized in Table 1 ▸.

A liquid-nitrogen-cooled double flat crystal monochromator was purchased from Bruker EST Inc. The first silicon (111) crystal diffracts the white beam into monochromatic photons ranging from 7 to 15 keV. To ensure good source properties, the monochromator was installed with a fixed exit. The second crystal is used to adjust the exit beam direction, so there is a height offset (25 mm) between the incidence and exit beams. The size of the X-ray beam at the entrance of the monochromator is typically 1.9 mm (H) × 1.2 mm (V). The monochromatic beam is allowed to ‘travel’ along the perpendicular and parallel directions of the second crystal as the Bragg angle is changed.

To ensure there is enough divergence between the two beams coming from the same straight section, one more deflection mirror was installed at 28.5 m from the source. The grazing angle of incidence is 3.8 mrad, which can broaden the distance between two beams horizontally.

The focusing element of the beamline is composed of two mirrors. One is the horizontal focusing mirror situated 31.2 m from the source. The other is the vertical focusing mirror situated 34 m downstream from the source. To have a balance between high photon flux and the reduction of high-order harmonic photons, the grazing-incidence angles were chosen carefully. For the BL19U2 beamline, the grazing angles are 3.5 mrad for both horizontal and vertical focusing mirrors, with a focusing ratio of 1:0.80 (horizontal) and 1:0.65 (vertical). Additionally, both focusing mirrors were coated with rhodium on the surface, which can significantly suppress the high-order harmonics. The focus of the monochromatic photons is on the detector plane. Typically, the beam is focused to 0.33 mm (H) × 0.05 mm (V) in the detector plane (22 m from the second focusing mirror), being 0.45 mm (H) × 0.11 mm (V) at the sample position (19.85 m from the second focusing mirror). The beam size in the sample plane can be further reduced at the expense of the photon flux.

### 2.2. Setup description   

**Figure 2:**
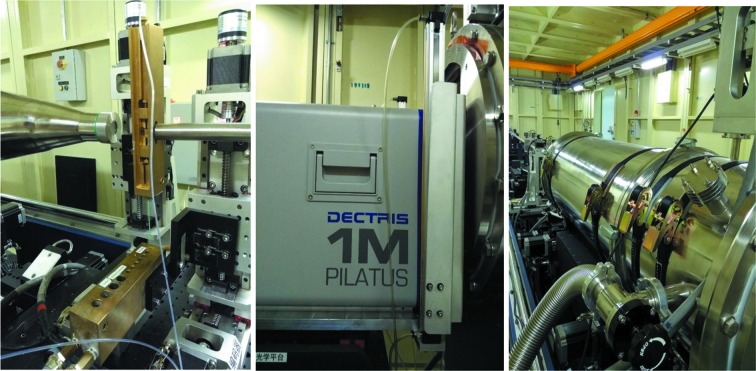
Experimental hutch for the BL19U2 BioSAXS beamline. Left: sample loading system with the exposure unit mounted at the flight tube entrance. Middle: flight tube exit (with aluminium alloy protective cover) and Pilatus 1M detector. Right: metal supporting table and motorized platforms.

The experimental hutch contains a 9.2 m-long metal table which supports the experimental hutch equipment (Fig. 2 ▸). The table has a cavity in the middle, in which four motorized supporting platforms were installed to facilitate the changing of the flight tube length. The flight tube itself is a modular design for variable length (0.5–7 m) and is mounted on motorized supporting platforms (*y* – lateral, *z* – vertical). Two pairs of scatterless guard slits (from Xenocs, Sassenage) are mounted right before the sample exposure unit. The evacuated flight tube houses a beamstop with a separate (*y*, *z*) motor control sitting immediately before the 150 µm-thick Kapton window. A high dynamic range pin-diode (S5971, Hamamatsu Photonics Co. Ltd) was buried in the beamstop to record the real-time transmitted-beam intensity. The two-dimensional detector (Pilatus 1M, Dectris Co. Ltd) with a protective tungsten cover is mounted on an independent motorized stage (*y* – lateral, *z* – vertical). It is possible to adjust the sample-to-detector distance for operation, on the basis of the sample characteristics, giving a momentum transfer range, *q* (*q* = 4π sin θ/λ, where 2θ is the scattering angle and λ is the wavelength), that varies in the range from 0.04 to 4.5 nm^−1^.

To meet the rapidly growing demands of crystallographers, biochemists and structural biologists, the BL19U2 beamline allows manual and automatic sample loading/unloading and data collection. In detail, a flow cell made of a cylindrical quartz capillary with a diameter of 1.5 mm and a wall thickness of 10 µm is used in the BL19U2 undulator SAXS beamline. This cell is embedded in a temperature-controlled copper holder block and sealed by O-rings. Two motorized *x*–*z* stages are employed to serve as the sample cell unit holder and sample storage plate. A Hamilton syringe pump (PSD4, Hamilton Robotics Co. Ltd) is used to pump samples and cleaning solutions into the cell. Typically, samples with a volume between 50 and 60 µl are delivered to the capillary for continuous-flow SAXS measurements, which can significantly reduce the effects of X-ray radiation damage. Automated loading of low sample volumes (10 µl) for static sample data collections and manual sample loading *via* syringe are also possible. The whole automated sample loading system has been designed to be easily changeable; therefore users can bring their own alternative sample handling equipment, for example, a stretching device to study fibre formation (Fig. 2 ▸).

In the BL19U2 BioSAXS beamline, samples are measured at different concentrations, typically between 0.5 and 10 mg ml^−1^, to detect interaction between the solutes. The sample storage plate accommodates PCR tubes (200 µl) for sample storage and 1.5 ml Eppendorf tubes for buffer storage. Users may perform either continuous-flow or static sample SAXS measurements and, if necessary, the syringe pump can be driven in reverse to recover samples after data acquisition. The sample flow rate is determined automatically by the amount of sample and by the total exposure time; typically the flow rate is set as 20 × 1 s exposures with a total of 20 s for each sample data acquisition. After the data collection, the sample is either flushed to waste or recovered, and this is followed by a subsequent automated washing cycle for cleaning of the sample capillary. The wash cycle includes a successive rapid-flow detergent–ethanol–water and MilliQ-water flush, followed by high-pressure drying with filtered air. The full cycle consisting of sample loading, data acquisition, capillary washing and drying takes approximately 3 min. The performance of this automated sample loading system is highly reliable during the operation of the BL19U2 BioSAXS beamline.

### 2.3. Beamline software   

**Figure 3:**
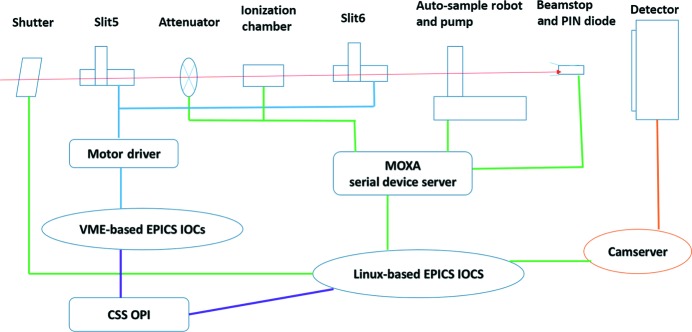
Schematic diagram of control and data acquisition system.

**Figure 4:**
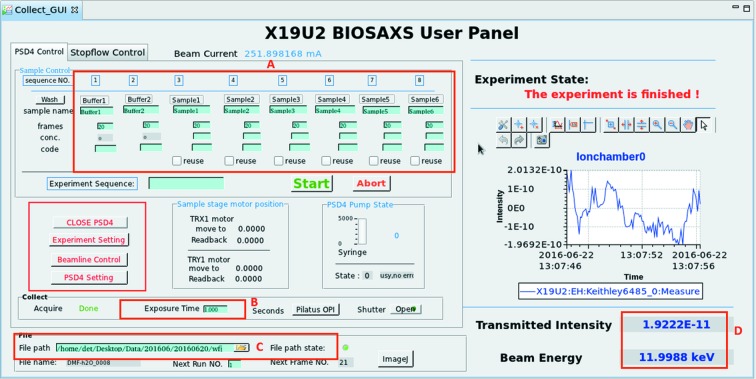
Beamline control and data acquisition software, allowing automated data acquisition. A, Sample description input; B, exposure time definition; C, image data saving path definition; D, storage ring real-time state illustration.

The beamline control and data acquisition software at the BL19U2 beamline follows the protocols developed by the SSRF, and the user interface is shown in Fig. 3 ▸. The software is composed of four parts: (i) motor movement control; (ii) automated sample loading system control; (iii) Pilatus 1M detector data collection control; and (iv) real-time transmitted-beam intensity recording. Standard *EPICS* development software was chosen to compile the control codes (http://www.aps.anl.gov/bcda/synApps/index.php), which easily combines the overall beamline device communication. VME racks, together with a MaxV8000 controller and the SSRF in-house-developed motor driver, are responsible for the end-station motor movement control. The control of the Pilatus detector is completely integrated into the SSRF *EPICS* software, which can simultaneously trigger data collection during sample exposure (Fig. 4 ▸). This software is completely adapted to the experimentalist’s needs with a user-friendly graphical user interface (GUI). This GUI displays several important beamline parameters such as the incident flux and the transmitted flux measured at the pin-diode mounted in the beamstop. Relevant parameters such as exposure time, image file name and sample description can also be set in the GUI and transmitted to the Pilatus 1M operating program, which triggers the data acquisition by opening the experimental shutter. Development of the GUI is based on CSS-BOY (see http://cs-studio.sourceforge.net/ for more details).

*ALBULA* 3.0 (Dectris Co. Ltd, Baden-Daettwil, Switzerland) is used to display the recorded two-dimensional images. Data processing can be performed either manually in batch using the software package *BioXTAS RAW* (Nielsen *et al.*, 2009 ▸) or automatically by running the EMBL (European Molecular Biology Laboratory), Hamburg, self-developed data analysis pipeline (*SASFLOW*; Franke *et al.*, 2012 ▸). This *SASFLOW* pipeline was integrated into the BL19U2 beamline in May 2015 with help from the BioSAXS group of the EMBL, Hamburg. Starting from handling two-dimensional scattering patterns and ending with the construction of the *ab initio* low-resolution particle shape, this *SASFLOW* pipeline is quite user friendly and dramatically improved the automation in the BL19U2 beamline. In addition, the full *ATSAS* software suite (Petoukhov *et al.*, 2007 ▸) is also available on the beamline for manual data processing.

### 2.4. Sample preparation laboratory   

**Figure 5:**
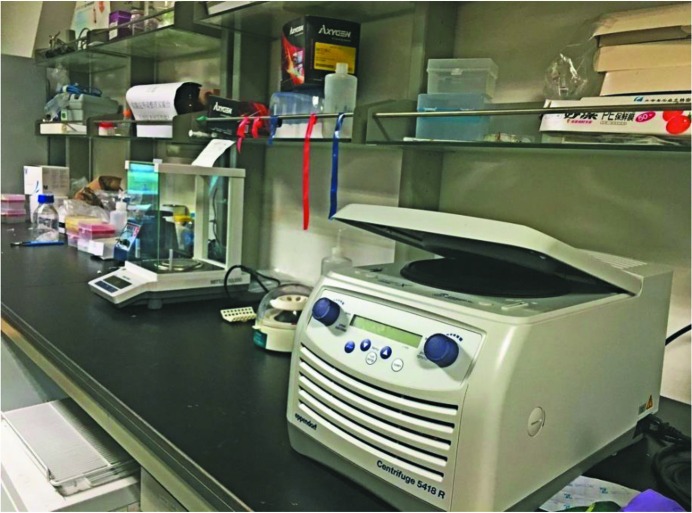
BL19U2 beamline sample preparation bench with the centrifuge, balance and other necessary biological sample preparation instruments.

A sample preparation room is shared by BL19U2 and three other crystallography beamlines located in the SSRF storage ring. There is one bench reserved for the use of the BioSAXS users (Fig. 5 ▸). This room is equipped with devices and instruments to meet the basic biological sample preparation requirements. A cooled mini-centrifuge (Eppendorf) is available for maintaining sample homogeneity. A nanodrop spectrophotometer is set to enable sample concentration measurements right before the experiment. Final sample preparation and characterization (*e.g.* dilutions, addition of ligands and additives) can be done prior to the SAXS measurement. If the users obtain negative feedback from the preliminary SAXS data analysis, they can immediately re-prepare the sample for measurement in our sample preparation laboratory.

## 3. Data example   

**Figure 6:**
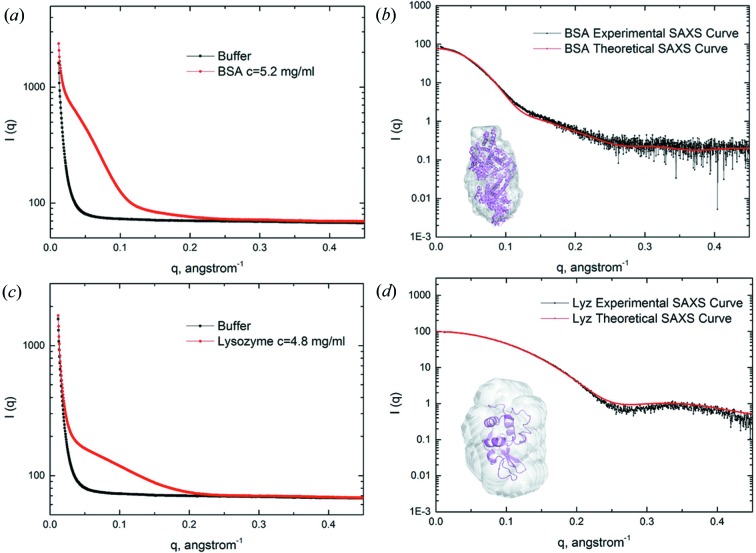
SAXS data collected on the BL19U2 beamline from BSA and LYS scaled for visualization. (*a*) Illustration of sample and background raw data for 5.2 mg ml^−1^ BSA. (*b*) Comparison of the experimental SAXS curve of BSA with the theoretical SAXS curve computed from the atomic structure (PDB: 3v03). (*c*) Illustration of sample and background raw data for 4.8 mg ml^−1^ LYS. (*d*) Comparison of the experimental SAXS curve of LYS with the theoretical SAXS curve computed from the atomic structure (PDB: 2lyz). Insets: *ab initio* models of BSA and LYS (grey) built from the respective SAXS experimental curves; the aligned atomic structure is represented in violet for comparison.

Bovine serum albumin (BSA) and lysozyme (LYS) samples of 60 µl were exposed to X-rays while flowing through the 1.5 mm-diameter quartz capillary using the automated sample loading system. The wavelength of X-ray radiation was set to 1.033 Å. The sample-to-detector distance was set such that the detecting range of momentum transfer *q* of the SAXS experiments was 0.01–0.45 Å^−1^. The samples were measured at a temperature of 293 K and the scattering curves of both BSA and LYS are shown in Figs. 6 ▸(*a*) and 6 ▸(*c*). The BSA averaged curve (from 20 frames of 1 s exposure each) with buffer scattering subtracted corresponds to 5.2 mg ml^−1^ solutions. The LYS averaged curve corresponds to 4.8 mg ml^−1^ solutions. Radii of gyration, *R*_g_, of 2.87 and 1.43 nm, respectively, were calculated. On the basis of the Guinier analysis, the forward-scattered intensity at zero angle *I*(0) corresponded to a molecular weight of 68 kDa and 15.2 kDa for BSA and LYS, respectively. The molecular weights were obtained by scaling the curves to the scattering of water in units of kDa as described by Orthaber *et al.* (2000 ▸). The discrepancy between the experimental scattering and the scattering calculated using *CRYSOL* (Svergun *et al.*, 1995 ▸) from the crystallographic structure of BSA (PDB entry 3v03; Majorek *et al.*, 2012 ▸) and LYS (PDB entry 2lyz; Diamond, 1974 ▸) yielded χ^2^ = 6.25 and χ^2^ = 2.95, respectively (Figs. 6 ▸ *b* and 6 ▸ *d*). These results strongly proved that the BL19U2 BioSAXS beamline can already collect reasonable SAXS data for biological macromolecules in solution.

## 4. Discussion and conclusions   

The newly constructed BL19U2 facility is the first one in China that can perform SAXS measurement in solution for biological macromolecules. It has the capability to perform up to 100 data collections on liquid protein samples per day with a high level of automation. Reliable and efficient data sets have been collected at this new facility. To ensure the competitiveness of the BioSAXS facility at the SSRF, future development will be focused on a variety of sample exposure environments for solution SAXS experiments, for example, reducing the sample volumes required for a single SAXS measurement and exploring the possibility of integrating a size-exclusion column into the beamline. In considering improvements in the field of SAXS data collecting detectors, the development of a time-resolved on-site device would also be in our future plan. As the number of proposals has increased rapidly for BL19U2 since its official opening to national users, we expect a corresponding rapid increase in the number of BioSAXS publications with data collected on BL19U2.

## Figures and Tables

**Table 1:** Beamline details

Beamline name	BL19U2
Source type	Undulator, U20
Mirrors	1.0 m-long Rh-coated focusing mirror, 3.5 mrad
	1.0 m-long Rh-coated focusing mirror, 3.5 mrad
Energy range (keV)	7–15
Wavelength range (Å)	0.82–1.77
Beam size (detector plane)	0.33 mm (H) × 0.05 mm (V)
Flux (photons s^−1^)	5 × 10^12^ (at 1.03 Å)
Sample loading	Robot
Cryo capability (K)	277–333
Sample cell unit	Quartz capillary
Detector type	CMOS hybrid pixel
Detector model	Pilatus 1M
*q* range (nm^−1^)	0.04–4.5 (at 1.03 Å)
